# Temporal patterns of sleep and eating among children during school closure in Japan due to COVID-19 pandemic: associations with lifestyle behaviours and dietary intake

**DOI:** 10.1017/S1368980022001148

**Published:** 2022-05-16

**Authors:** Minami Sugimoto, Kentaro Murakami, Satoshi Sasaki

**Affiliations:** 1 Institute for Future Initiatives, The University of Tokyo, Tokyo, Japan; 2 Department of Social and Preventive Epidemiology, School of Public Health, The University of Tokyo, 7-3-1 Hongo, Bunkyo-ku, Tokyo 113-0033, Japan; 3 Department of Social and Preventive Epidemiology, Division of Health Sciences and Nursing, Graduate School of Medicine, The University of Tokyo, Tokyo, Japan

**Keywords:** School closure, Temporal sleep and eating patterns, Lifestyle behaviours, Dietary intakes

## Abstract

**Objective:**

To identify temporal patterns of sleep and eating among school-age children during school closure due to the COVID-19 pandemic and to examine their associations with lifestyle behaviours and dietary intake.

**Design:**

In this cross-sectional study, questionnaires were used to assess sleep and eating times, lifestyle behaviours and dietary intake during school closure. Latent class analysis was performed to identify temporal patterns of sleep and eating based on self-reported clock times for wake-up, going to bed and eating meals. Lifestyle behaviours and dietary intake were compared between latent classes.

**Setting:**

Forty-eight primary and secondary schools in Japan.

**Participants:**

Totally, 6220 children (aged 8–15 years).

**Results:**

Four patterns, labelled ‘Very early (20 % of children)’, ‘Early (24 %)’, ‘Late (30 %)’ and ‘Very late (26 %),’ were identified and ordered according to the circadian timing. Latter patterns were characterised by later timings of sleep and eating, especially in clock times for wake-up, breakfast and lunch compared with earlier patterns. Children with latter patterns had a less physically active lifestyle, longer screen time (≥4 h/d), shorter study time (<2 h/d) and more frequent skipping of breakfast and lunch than those with earlier patterns. In addition, children with latter patterns had lower intakes of several vitamins, vegetables, fruits, fish and shellfish and dairy products and higher intakes of sugar and confectionaries and sweetened beverages.

**Conclusion:**

More than half of the participants had later wake-up, breakfast and lunch during school closure, which was associated with more unfavourable lifestyles and dietary intakes.

Under the global coronavirus disease 2019 (COVID-19) pandemic, school closure was conducted in many countries in 2020^(1)^. For example, almost all primary and secondary schools in Japan were closed from early March to the end of May. The school schedule is one of the major factors associated with the sleep and dietary habits of school-age children. For example, studies have consistently shown that school starting time is associated with sleep patterns^(2,3)^. In addition, previous studies have reported differences in sleep patterns^(4)^, physical activity^(5)^, time of eating breakfast^(6)^ and dietary intake^(7,8)^ between weekdays with school and weekends without school among children. Thus, the long-term school closure due to the pandemic might have altered sleep and dietary habits, including the time of sleep and eating among school-age children.

Recent studies concerning the influence of the COVID-19 pandemic have examined changes in lifestyle behaviours and dietary habits before and during school closure or lockdown among children. These studies mainly focused on screen time^(9,10)^, physical activity^(9–11)^, sleep habits^(10,12)^ and dietary habits^(11–13)^. However, to our knowledge, no study has investigated circadian rhythms of sleep and eating as well as their associations with lifestyle behaviours and dietary intake among children during long no-school days. In the context of an emerging focus on the whole dietary pattern^(14,15)^ and chrono-nutrition^(16)^, dietary intake should be examined at the level of specific eating occasions with the timing and distribution of daily eating^(14,17)^. Considering the circadian system of the human body^(16)^, the daily timing of eating should be captured in combination with sleep habits, which are associated with the time of eating meals^(18)^, breakfast skipping^(19,20)^, dietary intake^(4,20–23)^ and longer screen time^(23)^. Moreover, previous studies primarily assessed dietary variables, such as dietary intake^(12)^, eating frequency^(11)^ or diet quality scores^(13)^, with simple and/or non-validated questions. Quantitative assessments of dietary intake have rarely been conducted.

In a usual school year, Japanese school-aged children wake up early^(24,25)^ and eat school lunch^(8)^ regularly on weekdays. The absence of a school schedule and school lunch during school closure might alter the patterns of sleep and eating among children on weekdays. Further, irregular sleep and eating habits possibly resulted in unfavourable lifestyle behaviours among school-age children during school closure. The aim of this cross-sectional study was (i) to identify daily temporal patterns of sleep and eating among Japanese school-age children during school closure and (ii) to examine the associations between temporal patterns of sleep and eating and lifestyle behaviours and dietary intake.

## Methods

### Study design and participants

The target population of this cross-sectional survey was school-aged children (i.e. from third to sixth graders of elementary schools and from first to third graders of secondary schools; aged 8–15 years). First, we announced the conduct of the survey via a website, social media and direct e-mail to previous collaborators. As a result, forty-three elementary and secondary schools, one sports club and four research collaborators in fourteen out of forty-seven prefectures showed interest in the survey. Questionnaires were then distributed to school-age children by the schools, the sports club or research collaborators. The aim and procedure of the study were explained to children and their parents using a document attached to the questionnaires. The participants were considered to agree with participation in the survey when they answered and submitted the questionnaires. However, provided data from participants were excluded from the analysis when the participants indicated disagreement against using their data in the research even if they answered questionnaires. The present study was conducted according to the guidelines laid down in the Declaration of Helsinki. All procedures involving human subjects were approved by the Ethics Committee of the University of Tokyo, Faculty of Medicine (approval no. 2020056NI, 25 May 2020).

The information on the timing of school closure in the schools attended by the study participants was collected through the schools, sports club and research collaborators participating in this study. Although the date of closure started and reopened slightly differ by school or region, all schools attended by the participants started school closure in the first week of March 2020 and ended in the last week of May or the first week of June 2020. According to the Ministry of Education, Culture, Sports, Science and Technology in Japan, 99·0 % of all primary and secondary schools in Japan closed as of 16 March 2020^(26)^.

The survey was conducted immediately after schools reopened in June 2020. Participants were instructed to answer two types of questionnaires: a questionnaire for lifestyle behaviours and a brief-type self-administered diet history questionnaire for children and adolescents (BDHQ15y)^(27,28)^. Participants were asked to recall and answer their lifestyle behaviour and dietary intake in the previous month during school closure. Among 11 958 participants invited to the survey, 8512 participants (71 %) answered both questionnaires distributed in June 2020.

### Measurements

The questionnaire for lifestyle behaviours during school closure included questions on clock time for waking up and going to bed; frequency and clock time for eating breakfast, lunch, dinner and a late-night snack; the frequency of snacks; physical activity; screen time and study time. The participants were asked to recall and answer their lifestyle behaviour listed above in the previous month under school closure. In addition, parents were instructed to ask their children for their lifestyle if needed when answering the questionnaire. The questionnaire was answered by one of the family members of the participating children who mainly prepared meals at home. Table 1 summarises the analysed variables, the questions and/or methods used for measurement and generated categories for analysis. In brief, questions on clock times for waking up and going to bed were answered with units in h and min (hh:mm) and then categorised into <7:00, 7:00–7:59, 8:00–8:59 and ≥9:00 for wake-up time and <22:00, 22:00–22:59 and ≥23:00 for bedtime. Sleep duration was calculated using the wake-up time and bedtime. The midpoint of sleep was calculated as the midpoint between bedtime and wake-up time. Questions on the frequencies of eating breakfast, lunch, dinner and a late-night snack/week were answered in integer values from 0 to 7. Questions on clock times for eating breakfast, lunch, dinner and a late-night snack/week were answered in integer values from 0 to 24 h, as the clock time when the participants started to eat the concerned meal during school closure. Questions on snack frequency/d were answered in integer values, which were categorised into 0, 1, 2 and ≥3 times/d. Physical activity level (PAL) was calculated by dividing the metabolic equivalent-h score by 24 h. Metabolic equivalent-hour score was estimated by summing the self-reported time spent on each of a range of activities with various exercise intensities and metabolic equivalent value for each activity^(29,30)^. Participants with PAL < 1·40, 1·40–1·59, 1·60–1·89 and ≥1·9^(31)^ were categorised as inactive, low, middle and high, respectively. Questions on screen time and study time were answered using a nine-point scale; the answers of questions pertaining to both parameters were categorised into <2, 2–<4 and ≥4 h/d.

**Table 1:** Summary of analysed variables, questions and/or methods for measurement and generated category for analysis

Variables	Questions and/or method for assessment	Unit for answer (possible value range) or options	Generated category for analysis
Sleep habits			
Wake-up time	‘At what time did your child usually wake-up during school closure?’	h and min (0:01–24:00)	<6:59, 7:00–7:59, 8:00–8:59, and ≥9:00
Bedtime	‘What was the usual bedtime of your child during school closure?’	h and min (0:01–24:00)	<21:59, 22:00–22:59, and ≥23:00
Sleep duration	Calculated using self-reported wake-up time and bedtime	h and min (0:01–24:00)	<8:00, 8:00–9:59, 10:00–11:59, and ≥12:00
Midpoint of sleep	Calculated as the midpoint between the self-reported bedtime and wake-up time	h and min (0:01–24:00)	–
Dietary habits			
Frequency and clock time of eating meals	‘How many times/week and at what time did your child usually eat breakfast, lunch, dinner and a late-night snack during school closure? Here, breakfast, lunch, dinner, and a late-night snack were defined as meals including some staple foods with a high content in carbohydrates such as rice, bread, and noodles. Do not count any eating occasion without staple foods.’	For frequency, times/week (0 to 7 (integer)) For clock time for eating, time (hour) at which the child starts eating (0 to 24 (integer))	For clock time for eating, (for breakfast) <7:00, 7:00–7:59, 8:00–8:59, and ≥9:00; (for lunch) <12:00, 12:00–12:59, and ≥13:00; (for dinner) <19:00, 19:00–19:59, and ≥20:00; (for a late-night snack) <21 h and ≥21 h
Breakfast frequency	Categorised according to the self-reported frequency of eating breakfast/week	–	<7 (i.e. skipping at least once a week) and 7 times/week
Lunch frequency	Categorised according to the self-reported frequency of eating lunch/week	–	<7 (i.e. skipping at least once a week) and 7 times/week
A late-night snack frequency	Categorised according to the self-reported frequency of eating/week	–	0 and ≥ 1 time/week
Snack frequency	‘How many times did your children eat snacks other than meals (i.e. breakfast, lunch, dinner, and a late-night snack)/d during school closure? Do not count occasions wherein only drinks were taken.’	Times/d (≥0 (integer))	<2 and ≥2 times/d (for logistic analysis) 0, 1, 2, and ≥3 times/d (for chi-square test in Supplemental Table 2)
Lifestyle behaviour			
Physical activity level (PAL)	‘How long did your child do the following five activities (standing, walking, cycling, running, and other activities causing sweating) per day in the past month (during school closure)?’ PAL was calculated by dividing metabolic equivalent-hour score by 24 h. The metabolic equivalent-hour score was estimated by summing the self-reported time spent on each of a range of activities with various exercise intensities and metabolic equivalent value for each activity.*,†	Close to 0, 5 min, 15 min, 30 min, 45 min, 1 h, 1·5 h, 2 h, 3 h, 4 h (ten-point scale)	Inactive or low (PAL < 1·60), middle or high (PAL ≥ 1·60) (for logistic analysis) Inactive (PAL < 1·40), low (PAL = 1·40–1·59), middle (PAL = 1·60–1·89), high (PAL ≥ 1·9)‡ (for chi-square test in Supplemental Table 2)
Screen time	‘How long did your child spend a day watching a screen at home, such as playing computer games, watching television, or using a smartphone or tablet in an average week in the previous month (under school closure)?’	<30 min, 30 min–<1 h, 1 h–<2 h, 2 h–<3 h, 3 h–<4 h, 4 h–<5 h, 5 h–<6 h, 6 h–<7 h, ≥7h (nine-point scale)	<4, ≥4 h/d (for logistic analysis) <2, 2–<4, ≥4 h/d (for chi-square test in Supplemental Table 2)
Study time	‘How long did your child spend reading books and performing self-study at home/d during an average week in the previous month (under school closure)?’	<30 min, 30 min–<1 h, 1 h–<2 h, 2 h–<3 h, 3 h–<4 h, 4 h–<5 h, 5 h–<6 h, 6 h–<7 h, ≥7 h (nine-point scale)	<2, ≥2 h/d (for logistic analysis) <2, 2–<4, ≥4 h/d (for chi-square test in Supplemental Table 2)
Dietary intake			
Intakes of nutrients and foods	Assessed with a brief-type self-administered diet history questionnaire for children and adolescents	–	–
Binary variables for latent class analysis			
Wake-up time at 4–5, 6, 7, 8, 9, 10 and 11–15 h	Assigned ‘1’ (= event) or ‘2’ (= no-event) to indicate whether the participant woke up within each hour according to the self-reported wake-up time.	–	1 or 2
Bedtime at 19–20, 21, 22, 23, 24 and 1–5 h	Assigned ‘1’ (= event) or ‘2’ (= no-event) to indicate whether the participant went to bed within each hour according to the self-reported bedtime.	–	1 or 2
Eating meals at 5–6, 7, 8, 9, 10, 11, 12, 13, 14, 15, 16, 17, 18, 19, 20, 21 and 22–24 h	Assigned ‘1’ (= event) or ‘2’ (= no-event) to indicate whether the participant ate meals (breakfast, lunch, dinner or a late-night snack) within each hour according to the self-reported eating time when eating frequency was 5 times or more/week for a meal.	–	1 or 2

* Ainsworth BE, Haskell WL, Herrmann SD *et al.* (2011) Compendium of physical activities: a second update of codes and MET values. *Med Sci Sports Exerc* **43**, 1575–1581.

† Murakami K, Sasaki S, Okubo H *et al.* (2007) Association between dietary fiber, water and magnesium intake and functional constipation among young Japanese women. *Eur J Clin Nutr* **61**, 616–622.

‡ Ministry of Health Labour and Welfare (2020) Dietary reference intakes for Japanese. (in Japanese).

Dietary intake during school closure was assessed using the BDHQ15y, which was filled by participating children themselves, one of the children’s family members (who was mainly responsible for meal preparation), or children and their family members together. The participants were asked to answer their dietary habits in the previous month under school closure. The BDHQ15y is a four-page fixed-portion questionnaire concerning the consumption frequency of selected foods commonly consumed in Japan, general dietary behaviours and usual cooking methods during the previous month. The BDHQ15y was developed based on the sixteen-page comprehensive^(32)^ and four-page brief versions^(33,34)^ of a validated self-administered diet history questionnaire for Japanese adults. Estimates of daily intakes of foods (ninety food items in total), energy and selected nutrients were calculated using an ad hoc computer algorithm for the BDHQ15y based on the Standard Tables of Food Composition in Japan^(35)^ and sex-specific fixed portion size. The BDHQ15y was validated for selected nutrients, including protein, fatty acids and carotenoids, using biomarkers (erythrocyte fatty acid and serum carotenoid levels) as a gold standard^(27,28)^.

Date of birth, sex, body weight and height were self-reported as part of the BDHQ15y. Living status and sibling status were determined based on the relationship of family members living with the participating children asked with the questionnaire for lifestyle behaviours. Living status and sibling status were determined based on the relationship of family members living with the participating children for whom the questionnaire for lifestyle behaviours was filled. Living status was categorised into two groups: (i) living with both parents or (ii) living with a single parent and/or other relatives (including those living with a mother or father, a mother or father and/or other relatives and other relatives without both parents). Sibling status was categorised into two groups: (i) having at least one sibling at or under primary school age or (ii) not having any siblings at or under primary school age (i.e. not having any siblings or having an only sibling(s) over primary-school-age).

### Analysed participants

Participants who indicated disagreement in using their data (*n* 402) were excluded from the analysis. Furthermore, we excluded participants who were not in the targeted grades (*n* 88) and had missing information on the variables used (*n* 1763; *n* 1 for sex; *n* 3 for identification number; *n* 186 for frequency and eating time of breakfast, lunch or dinner; *n* 528 for snack frequency; *n* 773 for wake-up time or bedtime; *n* 378 for anthropometric data; *n* 10 for family structure, *n* 863 for physical activity; *n* 162 for screen time; *n* 78 for study time and *n* 3 for dietary data; some participants had more than one missing value).

**Fig. 1:**
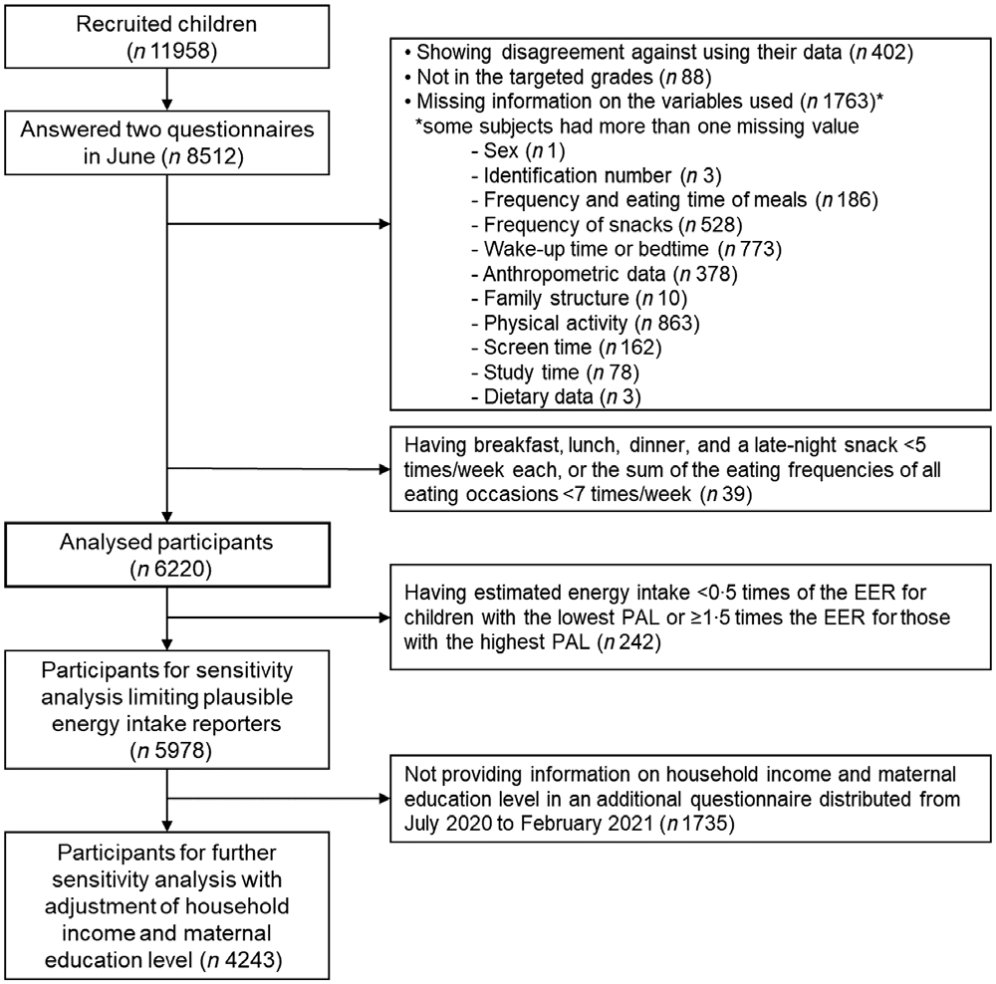
Flow chart of the participant for analysis. EER, estimated energy requirement, PAL, physical activity level. The thick line box shows the participants for the main analysis

To identify temporal patterns of sleep and eating with regard to the usual time of eating meals among participants, we excluded participants whose eating frequencies/week were less than five times for all eating occasions, or in whom the sum of the eating frequencies of all eating occasions was less than 7 times/week (*n* 39). For example, participants were excluded when they ate breakfast, lunch, dinner and a late-night snack 3, 4, 3 and 1 time(s)/week, respectively, or 0, 1, 5 and 0 time(s)/week, respectively. Finally, the analysed participants were 6220 children of 8512 potentially eligible participants who replied to the surveys in June 2020 (Fig. 1).

### Statistical analyses

#### Latent classes of temporal patterns of sleep and eating

**Fig. 2:**
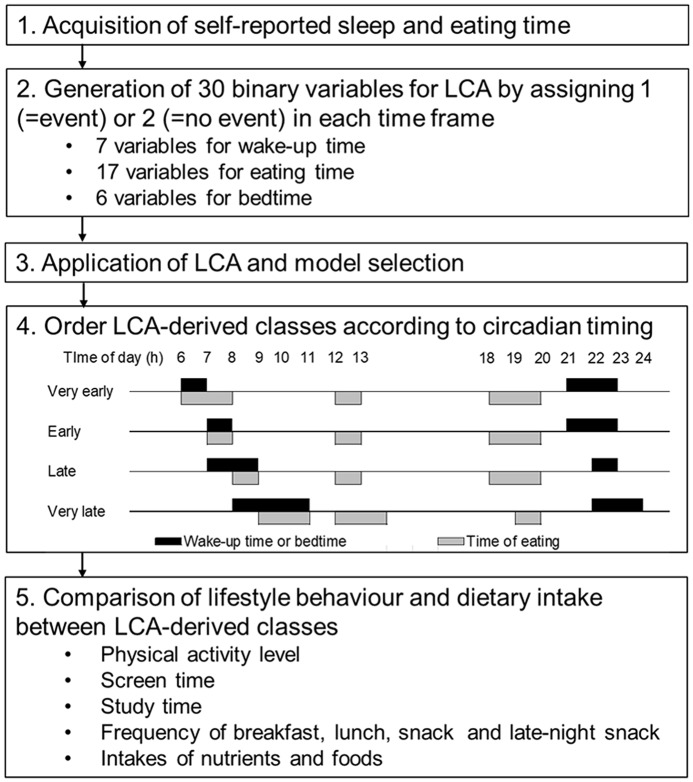
Summary of the analytical method. LCA, latent class analysis

Summary of the analytical method was shown in Fig. 2. Based on the clock time for wake-up; going to bed and eating breakfast, lunch, dinner and a late-night snack; binary variables (1 = event, 2 = no-event) were generated as input variables for latent class analysis (LCA). For clock time for wake-up and going to bed, binary variables indicated whether or not wake-up and going to bed had occurred within each hour of the day. For example, for the participants having clock time for wake-up at 7:45 am, a value of ‘1’ was assigned to the generated binary variable ‘wake-up time at 7 h’. A value of ‘2’ was assigned for the other binary variables for wake-up time. For the clock time of eating meals, binary variables indicated whether or not meal consumption had occurred within each hour of the day. To identify temporal eating patterns according to the usual clock time of eating meals, a value of ‘1’ was assigned when the eating frequency was 5 or more times/week for a particular meal; otherwise, a value of ‘2’ was assigned. Examples of variables generating eating time are shown in Supplemental Fig. 1. During the variables generating process, the self-classification of meals was not distinguished. For example, a value of ‘1’ was assigned to the binary variable ‘eating time at 11 h’ for participants who reported a clock time for eating breakfast at 11 am and those who reported a clock time for eating lunch at 11 am. Subsequently, generated binary variables with a few events were integrated to establish the number of input variables that gave the feasible solution in LCA. For example, going to bed from 1:00 am to 5:00 am was expressed in one binary variable, ‘bedtime at 1–5 h’. Binary variables with no events were excluded from the input variables for the LCA. Finally, thirty binary variables were generated: seven, six, and seventeen variables for wake-up time, bedtime and eating meals, respectively.

LCA was performed using SAS statistical software (version 9.4; SAS Institute Inc.) with the PLOC LCA procedure (version 1.3.2)^(36,37)^ to identify temporal patterns of sleep and eating. Models with two to six latent classes were tested. The Bayesian information criterion, Akaike information criterion (AIC), adjusted AIC and entropy were computed for each LCA. Models with lower Bayesian information criterion and AIC values suggested better goodness of fit. The four-class solution was chosen considering a combination of model fit and the interpretability of the classes^(37)^. The values of Bayesian information criterion, AIC and adjusted AIC decreased as the number of classes increased, but the decrease of the values levelled off among four, five and six classes (Table 2). Some patterns of five or six classes were less interpretable and resulted in classes representing <10 % of the sample (see online Supplemental Fig. 2). Thus, the four-class solution was better than the other solutions for further analysis.

**Table 2:** Model-fit indices for the latent class analysis model

Number of classes	Log likelihood	df	G2	AIC	BIC	Adjusted BIC	Entropy
2	−57 622·03	1073741762	48 400·84	48 522·84	48 933·70	48 739·86	0·90
3	−54 944·33	1073741731	43 045·44	43 229·44	43 849·10	43 556·75	0·97
4	−52 432·79	1073741700	38 022·35	38 268·35	39 096·82	38 705·96	0·98
5	−51 819·20	1073741669	36 795·18	37 103·18	38 140·45	37 651·08	0·98
6	−51 461·63	1073741638	36 080·03	36 450·03	37 696·11	37 108·23	0·99

AIC, the Akaike information criterion; BIC, Bayesian information criterion.

To interpret the identified patterns of sleep and eating, three or four peaks in the conditional probability of eating events were labelled as breakfast, lunch, dinner and a late-night snack, according to the chronological order in each latent class.

#### Associations between latent classes and socio-demographic and eating pattern indicators

All statistical analyses were performed using SAS 9.4 statistical software. All reported *P*-values were two-tailed, and a *P*-value < 0·05 was considered statistically significant. Data are presented as means and sd for continuous variables and as numbers and percentages for categorical variables. The mean (sd) clock times of eating meals were calculated for participants who ate each type of meal ≥5 times/week. Mean differences in continuous variables (i.e. clock times for wake-up, going to bed and eating, as well as basic characteristics) among LCA-derived classes were tested with linear regression models using the PROC GLM procedure. The ordinal scale (i.e. 1 to 4) according to the circadian timing was assigned for each class and used as a continuous variable in the linear regression models. Differences in categorical variables were tested using the *χ* ^2^ test.

The risks of classifying participants as having unfavourable lifestyles were tested using logistic regression. Examined unfavourable lifestyles were as follows: physical inactivity or low PAL; longer screen time (4 h or more/d); shorter study time (less than 2 h/d); skipping breakfast at least once/week; skipping lunch at least once/week; having a late-night snack at least once/week and having a snack at least twice/d. First, crude odds ratios (OR) and 95 % CI for the risk of having unfavourable lifestyles were calculated for each LCA-derived class; the LCA-derived class having the earliest circadian timing was used as the reference. Thereafter, multivariate-adjusted OR and 95 % CI were calculated by entering the following confounding factors into the regression model: age, sex, living status and sibling status.

Linear regression models were constructed to examine the association between LCA-derived classes and intakes of nutrient and food using the ordinal scale of LCA-derived classes as a continuous variable. A multivariate-adjusted model analysis was also performed with age, sex, living status and sibling status as confounding variables.

### Sensitivity analysis

Sensitivity analysis was performed only on participants with more plausible reported energy intake (*n* 5978) (Fig. 1). Participants were excluded if their energy intake estimated using the BDHQ15y was <0·5 times the estimated energy requirement for children with the lowest PAL or ≥1·5 times the estimated energy requirement for those with the highest PAL. Further sensitivity analysis was performed with adjustments for household income and maternal education level (*n* 4243) (Fig. 1). Information regarding household income and maternal education level was collected with an additional questionnaire distributed to the participants several months after the first survey, from July 2020 to February 2021. Participants who did not answer the additional questionnaire were excluded from the analysis.

## Results

The mean (sd) age, height and weight of participants were 11·0 (1·9) years, 144·8 (12·5) cm and 38·4 (10·7) kg, respectively. Fifty-one percent of the participants were boys and 67 % were primary school-aged.

**Fig. 3:**
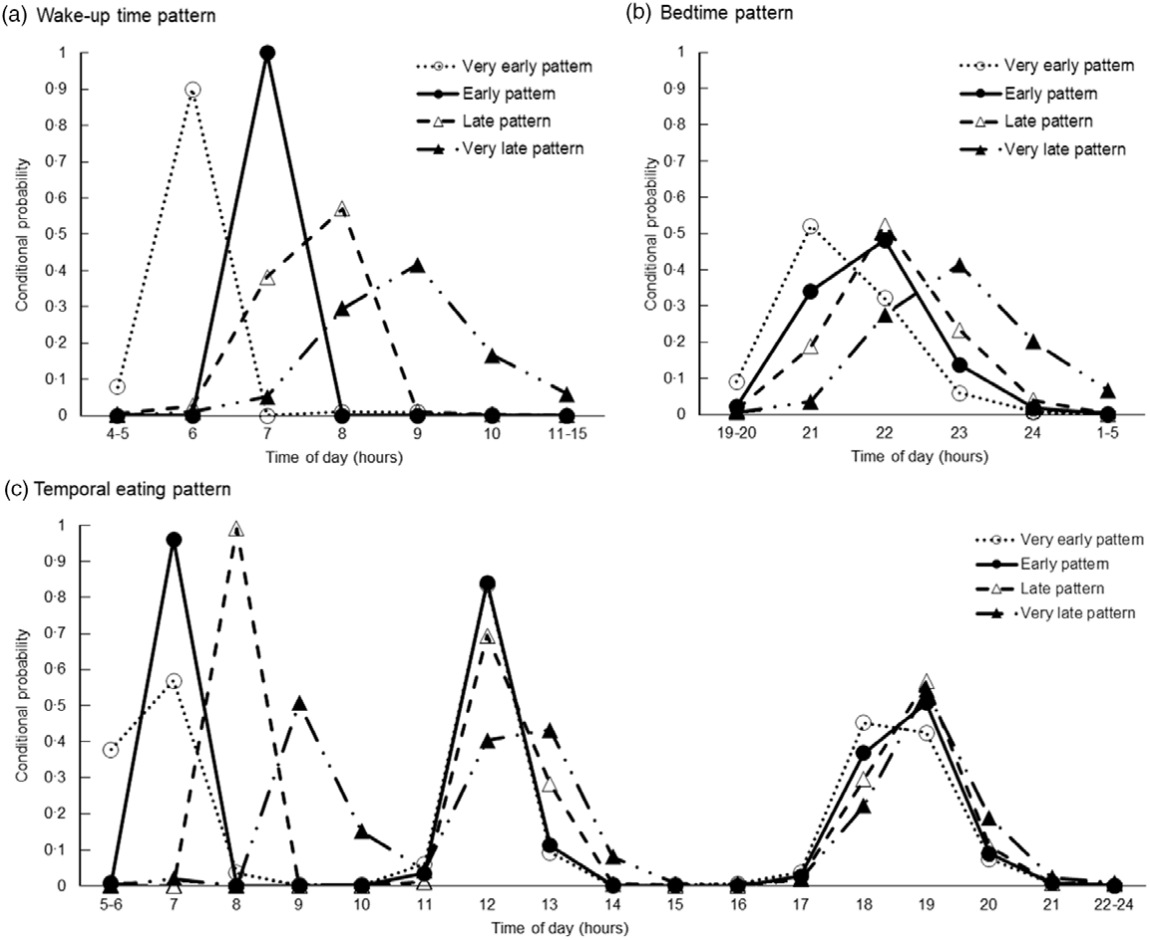
Conditional probabilities of: (a) wake-up time; (b) bedtime and (c) eating time across the day according to latent class analysis-derived temporal patterns of sleeping and eating among 6220 school-aged children. Dashed lines with a white circle represent the ‘Very early’ pattern, solid lines with a black circle represent the ‘Early’ pattern, dashed lines with a white triangle represent the ‘Late’ pattern, and dashed lines with a black triangle represent the ‘Very late’ pattern

Using LCA, four temporal patterns of sleep and eating were derived. Each class was labelled based on distinguishing features, as shown by high or low conditional probability for wake-up, going to bed and consuming a meal at each time frame of a usual day during school closure. Figure 3 shows the confidence probabilities for each pattern. The first pattern was labelled ‘Very early’, as participants in this class (20 % of participants) woke up and ate breakfast earlier than those in the other classes. The second pattern was labelled ‘Early’, as participants in this class (24 % of participants) woke up and ate breakfast 1 h later than those in the first pattern but earlier than those in the other two patterns. The third pattern was labelled ‘Late’, as participants in this pattern (30 % of participants) woke up and ate breakfast 1–2 h later than those in the first pattern. Peaks in the conditional probability of eating lunch at 12 pm were similar among the ‘Very early,’ ‘Early’ and ‘Late’ patterns. The fourth pattern labelled ‘Very late’ (26 % of participants) was characterised by participants with the latest timings of wake-up and eating breakfast. Latter patterns were also characterised by participants with later timings of eating dinner or a late-night snack and bedtime. However, differences in the timings of eating dinner or a late-night snack and bedtime were less distinctive among classes compared with those of wake-up and eating breakfast.

Among all participants, the mean (sd) clock times were 7:39 (1:10) for wake-up and 22:18 (1:01) for going to bed (Table 3). The mean (sd) sleep duration was 9:21 (0:58), and the midpoint of sleep was 2:58 (0:58). The mean (sd) clock times for each eating occasion were 8 (1) h (*n* 5650) for breakfast, 12 (1) (*n* 6108) h for lunch, 19 (1) h (*n* 6180) for dinner and 20 (1) h (*n* 69) for a late-night snack, among participants eating the concerned meals ≥5 times/week. The clock times for wake-up, going to bed and eating meals, as well as the midpoint of sleep were later for children with latter patterns than for those in earlier patterns (*P* < 0·0001). Sleep duration was longer in participants with the latter patterns than those with earlier patterns (*P* < 0·0001).

**Table 3:** Sleep habits and clock time for eating in 6220 school-age children (third to sixth grade of primary school and first to third grade of secondary school) according to latent class analysis-derived temporal patterns of sleep and eating

	All (*n* 6220)				‘Very early’ pattern (*n* 1219)				‘Early’ pattern (*n* 1503)				“Late” pattern (*n* 1868)				‘Very late’ pattern (*n* 1630)				*P* *
	*n*	%	Mean	SD	*n*	%	Mean	SD	*n*	%	Mean	SD	*n*	%	Mean	SD	*n*	%	Mean	SD	
Sleep habits																					
Clock time for wake-up (hh:mm)			7:39	1:10			6:19	0:29			7:09	0:13			7:44	0:34			9:00	1:04	<0·0001
<7:00	1280	21			1197	98			0	0			65	3			18	1			<0·0001
7:00–7:59	2297	37			0	0			1503	100			713	38			81	5			
8:00–8:59	1562	25			11	1			0	0			1060	57			491	30			
≥9:00	1081	17			11	1			0	0			30	2			1040	64			
Clock time for going to bed (hh:mm)			22:18	1:01			21:35	0:44			22:00	0:45			22:17	0:46			23:09	1:04	<0·0001
<21:59	1738	28			754	62			543	36			373	20			68	4			<0·0001
22:00–22:59	2543	41			384	32			722	48			980	52			457	28			
≥23:00	1939	31			81	7			238	16			515	28			1105	68			
Sleep duration (hh:mm)			9:21	0:58			8:44	0:51			9:09	0:45			9:27	0:50			9:51	1:05	<0·0001
<8:00	271	4			139	11			44	3			40	2			48	3			<0·0001
8:00–9:59	3856	62			1002	82			1132	75			1130	60			592	36			
10:00–11:59	1996	32			65	5			326	22			682	37			923	57			
≥12:00	97	2			13	1			1	0			16	1			67	4			
Midpoint of sleep (hh:mm)			2:58	0:58			1:57	0:27			2:34	0:24			3:01	0:32			4:04	0:55	<0·0001
Clock time for eating†,‡																					
Breakfast (clock time of day, hour)	5650	100	8	1	1204	100	7	1	1451	100	7	0	1866	100	8	0	1129	100	9	1	<0·0001
<7:00	477	8			464	39			12	1			0	0			1	0			<0·0001
7:00–7:59	2170	38			699	58			1439	99			0	0			32	3			
8:00–8:59	1904	34			38	3			0	0			1866	100			0	0			
≥9:00	1099	19			3	0			0	0			0	0			1096	97			
Lunch (clock time of day, hour)	6108	100	12	1	1209	100	12	0	1492	100	12	0	1856	100	12	1	1551	100	13	1	<0·0001
<12:00	194	3			76	6			52	3			22	1			44	3			<0·0001
12:00–12:59	4239	68			1018	84			1266	85			1294	70			658	42			
≥13:00	1675	27			115	10			174	12			537	29			849	55			
Dinner (clock time of day, hour)	6180	100	19	1	1213	100	19	1	1496	100	19	1	1860	100	19	1	1611	100	19	1	<0·0001
<19:00	2180	35			607	50			590	39			586	32			397	25			<0·0001
19:00–19:59	3220	52			513	42			762	51			1057	57			888	55			
≥20:00	780	13			93	8			144	10			217	12			326	20			
Late-night snack (clock time of day, hour)	69	100	20	1	9	100	20	1	9	100	19	1	17	100	20	2	34	100	21	1	0·0001
<21:00	37	54			7	78			8	89			11	65			11	32			0·003
≥21:00	32	46			2	22			1	11			6	35			23	68			

* For continuous variables, *P* values represent *P*_for trend_ in temporal patterns of sleep and eating. For categorical variables, *P* values were tested using the *χ* ^2^ test. The trend of association was examined using a linear regression model with the ordinal scale of temporal patterns of sleep and eating (1 = ‘Very early’ pattern, 2 = ‘Early’ pattern, 3 = ‘Late’ pattern and 4 = ‘Very late’ pattern) as a continuous variable.

† In the questionnaire, the clock time for eating was answered with an integer value of hour by participants (e.g. breakfast consumed between 7:00 and 7:59 was answered as ‘7’).

‡ Participants who ate breakfast, lunch, dinner or late-night snacks less than 5 times/week were excluded from the analysis.

Participants with latter patterns tended to be older and were predominantly girls (Table 4). The proportion of participants living with a single parent and/or other relatives and having siblings aged at or under primary school age was higher in the latter patterns (*P* < 0·0001). Latter patterns had higher proportions of participants who were inactive or had a low PAL (*P* < 0·0001), had a screen time of 4 h or more (*P* < 0·001), had a study time of less than 2 h (*P* < 0·0001), skipped breakfast (*P* < 0·001) and lunch (*P* < 0·0001), had a late-night snack at least once/week (*P* < 0·001) and had snacks at least twice/week (*P* = 0·001) (Table 4 and see online Supplemental Table 2).

**Table 4:** Characteristics of 6220 school-aged children (third to sixth grade of primary school and first to third grade of secondary school) according to latent class analysis-derived temporal patterns of sleeping and eating

	All (*n* 6220)		‘Very early’ pattern (*n* 1219)		‘Early’ pattern (*n* 1503)		‘Late’ pattern (*n* 1868)		‘Very late’ pattern (*n* 1630)		*P* *
	Mean	sd	Mean	sd	Mean	sd	Mean	sd	Mean	sd	
Age (years)	11·0	1·9	10·6	1·8	10·8	1·8	10·9	1·8	11·6	1·9	<0·0001
Height (cm)	144·8	12·5	142·5	12·0	143·5	12·1	144·4	12·4	147·9	12·6	<0·0001
Weight (kg)	38·4	10·7	36·7	10·0	37·3	10·1	38·1	11·0	41·1	11·0	<0·0001
	*n*	%	*n*	%	*n*	%	*n*	%	*n*	%	
Sex (boys)	3158	51	692	57	767	51	930	50	769	47	<0·0001
Grade											
Primary school	4137	67	896	74	1086	72	1298	69	857	53	<0·0001
Secondary school	2083	33	323	26	417	28	570	31	773	47	
Family structure											
Living with both parents	5459	88	1082	89	1341	89·2	1686	90·3	1350	82·8	<0·0001
Living with single parent and/or other relatives†	761	12	137	11	162	10·8	182	9·7	280	17·2	
Sibling status: having sibling(s) aged at or under primary-school-age											<0·0001
Yes	3048	49	501	41·1	716	47·6	882	47·2	949	58·2	
No	3172	51	718	58·9	787	52·4	986	52·8	681	41·8	
Physical activity level‡											<0·0001
Inactive or low	3865	62	674	55	908	60	1158	62	1125	69	
Middle or high	2355	38	545	45	595	40	710	38	505	31	
Screen time/d§											<0·0001
<4 h	3604	58	840	69	1011	67	1119	60	634	39	
≥4 h	2616	42	379	31	492	33	749	40	996	61	
Study time/d║											<0·0001
<2 h	3374	54	585	48	724	48	970	52	1095	67	
≥2 h	2846	46	634	52	779	52	898	48	535	33	
Breakfast frequency											<0·0001
Skipping ≥1 times/week	1013	16	60	5	112	7	151	8	690	42	
Daily	5207	84	1159	95	1391	93	1717	92	940	58	
Lunch frequency (times/week)											<0·0001
Skipping ≥1 times/week	381	6	43	4	52	3	73	4	213	13	
Daily	5839	94	1176	96	1451	97	1795	96	1417	87	
A late-night snack frequency (times/week)¶											<0·0001
0	5847	94	1186	97	1447	96	1759	94	1455	89	
≥ 1	373	6	33	3	56	4	109	6	175	11	
Snack frequency/d											0·001
0–1	3728	60	742	61	918	61	1158	62	910	56	
≥ 2	2492	40	477	39	585	39	710	38	720	44	

* For continuous variables, *P* values represent *P*_for trend_ in temporal patterns of sleep and eating. For categorical variables, *P* values were tested using the *χ* ^2^ test. The trend of association was examined using a linear regression model with the ordinal scale of temporal patterns of sleeping and eating (1 = ‘Very early’ pattern, 2 = ‘Early’ pattern, 3 = ‘Late’ pattern and 4 = ‘Very late’ pattern) as a continuous variable.

† Including participants living with mother (or father), living with mother (or father) and/or other relatives (e.g. living with mother and grandparents) and living with other relatives without both parents (e.g. living with grandparents).

‡ Participants with physical activity level (PAL) <1·60^(31)^ were categorised as ‘inactive or low PAL.’ PAL was calculated by dividing the metabolic equivalent-hour score by 24 h. The metabolic equivalent-hour score was estimated by summing the product of the time spent on each of a range of activities (sleeping, standing, walking, cycling, running and other activities causing sweating) with various exercise intensities and metabolic equivalent values for each activity^(29,30)^.

§ Screen time included the time spent in watching television; using a computer, smartphone or tablet and playing video games.

║ Study time included time spent reading books and self-studying.

¶ A late-night snack was defined as a meal including staple foods (i.e. rice, bread or noodles).

The risk of having unfavourable lifestyle behaviours was high in participants with latter patterns compared with those with the ‘Very early’ pattern (all *P*_for trend_ <0·0001) (Table 5). The results were similar after adjustment for confounding factors (all *P*_for trend_ <0·0001, except for having snacks ≥2 times/d (*P*_for trend_ was 0·003)). Compared with the ‘Early’ and ‘Late’ patterns, the ‘Very late’ pattern had much higher OR for longer screen time (adjusted OR = 3·13, 95 % CI (2·66, 3·68)), skipping breakfast (adjusted OR = 12·28, 95 % CI (9·28, 16·26)), skipping lunch (adjusted OR = 3·71, 95 % CI (2·63, 5·12)) and having a late-night snack (adjusted OR = 3·86, 95 % CI (2·62, 5·69)).

**Table 5:** Odds ratios for unfavourable lifestyle and dietary habits according to latent class analysis-derived temporal patterns of sleeping and eating among 6220 school-age children (third to sixth grade of primary school and first to third grade of secondary school)

		‘Very early’ pattern (*n* 1219)			‘Early’ pattern (*n* 1503)				‘Late’ pattern (*n* 1868)				‘Very late’ pattern (*n* 1630)				*P*_for trend_ *
		*n*	%		*n*	%	OR	95 % CI	*n*	%	OR	95 % CI	*n*	%	OR	95 % CI	
Inactive or low physical activity level†	Crude	674	55	Ref	908	60	1·23	1·06, 1·44	1158	62	1·32	1·14, 1·53	1125	69	1·80	1·54, 2·10	<0·0001
	Adjusted‡			Ref			1·19	1·02, 1·39			1·27	1·09, 1·47			1·63	1·39, 1·91	<0·0001
Longer screen time (≥4 h/d)	Crude	379	31	Ref	492	33	1·08	0·92, 1·27	749	40	1·48	1·27, 1·73	996	61	3·48	2·98, 4·07	<0·0001
	Adjusted‡			Ref			1·07	0·91, 1·27			1·47	1·26, 1·72			3·13	2·66, 3·68	<0·0001
Short study time (<2 h/d)	Crude	585	48	Ref	724	48	1·01	0·87, 1·17	970	52	1·17	1·01, 1·35	1095	67	2·22	1·90, 2·58	<0·0001
	Adjusted‡			Ref			1·04	0·89, 1·21			1·23	1·06, 1·43			2·47	2·11, 2·89	<0·0001
Skipping breakfast ≥1 times/week	Crude	60	5	Ref	112	7	1·56	1·13, 2·15	151	8	1·70	1·25, 2·31	690	42	14·18	10·74, 18·71	<0·0001
	Adjusted‡			Ref			1·52	1·10, 2·10			1·64	1·20, 2·23			12·28	9·28, 16·26	<0·0001
Skipping lunch ≥1 times/week	Crude	43	4	Ref	52	3	0·98	0·65, 1·48	73	4	1·11	0·76, 1·63	213	13	4·11	2·94, 5·76	<0·0001
	Adjusted‡			Ref			0·96	0·63, 1·44			1·08	0·74, 1·59			3·71	2·63, 5·23	<0·0001
Having late-night snack ≥1 times/week	Crude	33	3	Ref	56	4	1·39	0·90, 2·15	109	6	2·23	1·50, 3·31	175	11	4·32	2·96, 6·32	<0·0001
	Adjusted‡			Ref			1·42	0·91, 2·20			2·25	1·51, 3·35			3·86	2·62, 5·69	<0·0001
Having snack ≥2 times/d	Crude	477	39	Ref	575	38	0·99	0·85, 1·16	710	38	0·95	0·82, 1·11	720	44	1·23	1·06, 1·43	<0·0001
	Adjusted‡			Ref			1·00	0·86, 1·17			0·96	0·83, 1·12			1·28	1·10, 1·50	0·003

* Logistic regression models were used with the ordinal scale of temporal patterns of sleeping and eating (1 = ‘Very early’ pattern, 2 = ‘Early’ pattern, 3 = ‘Late’ pattern and 4 = ‘Very late’ pattern) as a continuous variable.

† Participants with physical activity level (PAL) <1·60^(31)^ were categorised as ‘Inactive or low PAL.’ PAL was calculated by dividing the metabolic equivalent-hour score by 24 h. The metabolic equivalent-hour score was estimated by summing the product of the time spent on each of a range of activities (sleeping, standing, walking, cycling, running and other activities causing sweating) with various exercise intensities and metabolic equivalent values for each activity^(29,30)^.

‡ In the adjusted model, sex, age, living status and sibling status were adjusted.

Participants with latter patterns had lower intakes for protein; dietary fibre; vitamins A, C, B_6_ and B_12_; thiamine; riboflavin; niacin; folate; potassium; Ca; Mg; Fe; pulses; vegetables; fruits; fish and shellfish and dairy products and higher intakes of carbohydrate, sugars and confectionaries and sweetened beverages compared with those with earlier patterns (all *P*_for trend_ < 0·0001 except for carbohydrates) (Table 6). Dietary intakes did not significantly differ among participants with different patterns for total fat, saturated fat, Na and meat. After adjusting for confounding factors, the direction of association between LCA-derived classes and dietary intake did not change (see online Supplemental Table 2). The statistical significance of *P*_for trend_ for cereal intake disappeared after the adjustment, but the results of other nutrients and foods did not change before and after the adjustment.

**Table 6:** Dietary intakes according to latent class analysis-derived temporal patterns of sleep and eating among 6220 school-age children (3rd to 6th grade of primary school and 1st to 3rd grade of secondary school)

	All (*n* 6220)		“Very early” pattern (*n* 1219)		“Early” pattern (*n* 1503)		“Late” pattern (*n* 1868)		“Very late” pattern (*n* 1630)		*P* for trend*
	Mean	sd	Mean	sd	Mean	sd	Mean	sd	Mean	sd	
Nutrient intake											
Protein (% of energy)	13·9	2·2	14·4	2·3	14·1	2·2	13·9	2·1	13·5	2·3	<0·0001
Total fat (% of energy)	30·8	5·5	30·8	5·4	31·1	5·6	30·7	5·4	30·8	5·4	0·23
SFA (% of energy)	10·1	2·4	10·1	2·4	10·2	2·5	10·1	2·4	10·1	2·5	0·29
Carbohydrate (% of energy)	53·7	6·4	53·4	6·3	53·3	6·6	53·9	6·3	54·2	6·5	0·0007
Dietary fibre (g/4184 kJ)	5·7	1·6	5·9	1·7	5·9	1·6	5·8	1·5	5·5	1·5	<0·0001
Vitamin A (μg retinol equivalents/4184 kJ)	313	175	333	212	320	137	315	186	288	157	<0·0001
Thiamine (mg/4184 kJ)	0·4	0·1	0·41	0·08	0·40	0·07	0·40	0·07	0·39	0·07	<0·0001
Riboflavin (mg/4184 kJ)	0·7	0·2	0·75	0·18	0·73	0·17	0·71	0·17	0·68	0·18	<0·0001
Niacin (mg/4184 kJ)	7·0	1·7	7·2	1·8	7·1	1·7	7·1	1·7	6·8	1·8	<0·0001
Vitamin B_6_ (mg/4184 kJ)	0·6	0·1	0·58	0·14	0·57	0·13	0·56	0·13	0·54	0·13	<0·0001
Vitamin B_12_ (μg/4184 kJ)	3·4	1·7	3·5	1·8	3·4	1·5	3·4	1·6	3·2	1·7	<0·0001
Folate (μg/4184 kJ)	160	53	168	55	165	53	161	52	149	52	<0·0001
Vitamin C (mg/4184 kJ)	54·1	22·9	55·7	22·6	55·5	22·7	54·9	22·6	50·5	23·4	<0·0001
Na (mg/4184 kJ)	1901	404	1906	402	1910	405	1905	395	1883	415	0·22
Potassium (mg/4184 kJ)	1168	274	1218	287	1191	270	1167	264	1112	272	<0·0001
Ca (mg/4184 kJ)	322	108	344	114	330	104	318	104	304	108	<0·0001
Mg (mg/4184 kJ)	119	23	123	25	121	23	119	22	114	22	<0·0001
Fe (mg/4184 kJ)	3·8	0·9	4·0	0·9	3·9	0·8	3·8	0·8	3·7	0·8	<0·0001
Food intake (g/4184 kJ)											
Cereal	220	64	220	63	216	62	222	64	220	65	0·048
Sugars and confectioneries	52·3	28·2	49·7	28·3	52·4	28·1	51·9	27·0	54·5	29·4	0·0001
Pulses	25·3	17·4	26·9	18·1	26·6	17·5	25·3	17·2	22·8	16·5	<0·0001
Vegetables	108	58	117	63	113	58	109	56	97	57	<0·0001
Fruits	28·2	28·4	29·8	29·2	30·0	28·3	28·7	28·1	24·7	27·9	<0·0001
Fish and shellfish	27·2	15·6	28·6	15·7	27·9	15·4	27·3	15·4	25·4	16·0	<0·0001
Meat	39·5	16·9	39·8	17·5	39·9	16·9	39·3	16·0	39·1	17·3	0·52
Dairy products	104	88	118	92	106	84	100	85	94	90	<0·0001
Sweetened beverages	42·5	68·8	30·3	50·7	38·2	64·3	39·2	60·3	59·1	87·9	<0·0001

* Trend of association was examined using a linear regression model with the ordinal scale of temporal patterns of sleeping and eating (1 = ‘Very early’ pattern, 2 = ‘Early’ pattern, 3 = ‘Late’ pattern and 4 = ‘Very late’ pattern) as a continuous variable.

Sensitivity analyses limiting analysed participants with reported energy intake and a further adjustment of household income and maternal education level did not considerably alter the abovementioned findings.

## Discussion

To our knowledge, this is the first study that explored temporal patterns of sleep and eating and examined their associations with lifestyle behaviours and dietary intake. In addition, no study has identified temporal eating patterns among school-age children during school closure in any country including Japan. Inconsistent with the findings of a previous study wherein three eating patterns (including irregular eating) were identified among Australian adults using LCA^(17)^ participants with all four identified patterns in our study had three main meals/d at a regular time. This inconsistency with the previous study might be because we did not include the time of eating snacks in the LCA in this study. Considering the higher snack frequency among participants with the ‘Very late’ pattern than among participants with the other three patterns, different temporal eating patterns might be identified when the time of eating snacks is considered.

Among participants with the four identified patterns, those classified as having ‘Late’ and ‘Very late’ patterns waked up on average at 7:44 am and 9:00 am, respectively. On weekdays during usual school days, Japanese school-age children wake up between 6 am and 7 am on average^(24,25)^. Thus, children with ‘Late’ and ‘Very late’ patterns woke up and ate breakfast much later than they did during usual school days on average, whereas children with ‘Very early’ and ‘Early’ patterns kept their time schedule as they did during usual school days. However, dinner time and bedtime were not as distinctively different as wake-up and breakfast times between participants with different patterns. A possible reason for the less distinctive difference in dinner time and bedtime between participants with different patterns is the frequency of family eating. A previous study reported a higher frequency of family eating at dinner compared with that at breakfast^(38)^. Having family meals at breakfast or dinner might play a role in preventing a delay in eating time, which in turn prevents delays in wake-up time or bedtime. Although the frequency of family eating at each meal was not evaluated among participants in this study, it is possible that participants with earlier patterns frequently had family eating at both breakfast and dinner, whereas those with latter patterns frequently had family meals at dinner but not at breakfast. Consequently, the time of dinner and bedtime would not be delayed and would less distinctively differ between participants with different patterns. In contrast, a lower frequency of family eating at breakfast could delay wake-up and breakfast times in participants with latter patterns, possibly because the management of wake-up and breakfast times highly depends on children themselves.

Regarding sleep habits among participants, children with ‘Late’ and ‘Very late’ patterns might have later chronotypes because they had a later time of midpoint sleep. Previous studies reported associations between later chronotype or later midpoint sleep and longer screen time^(23)^, a higher frequency of skipping meals^(19,20)^, consuming food at late hours^(18)^, a lower intake of fruits and/or vegetables^(4,21,23)^ and a higher intake of soft drinks^(21,23)^ or lower diet quality^(22)^. Thus, our study findings are consistent with those of previous studies. A higher frequency of skipping meals and consuming snacks might have resulted in poor dietary intake (such as lower intakes of vegetables and fruits, as well as higher intakes of sugar, confectionaries and sweetened beverages) among participants with latter patterns. Considering the larger social jet lag among children with later chronotypes^(39)^ and the associations between school time and time for sleep^(2,3)^ and breakfast^(6)^, children with later chronotypes could be more vulnerable to changes in their circadian timing during no-school days.

A possible reason for the association of latter temporal patterns of sleep and eating with unfavourable lifestyles in the present study was the family environment, including parenting practice. Previous studies have shown an association between parenting practice and screen time^(40)^, PAL^(40)^ and sleep habits^(41)^ among children. Children with latter patterns were possibly less likely to have family rules regarding screen time and sleep habits and less likely to be encouraged to engage in physical activity. During school closure, managing children’s lifestyles might depend highly on their family environment and caregivers. Mothers reportedly have more childcare and household work during lockdown periods than fathers^(42,43)^. In Japan, working mothers with primary school-age children are more likely to work from home, unlike fathers with primary school-age children and parents with secondary school-age children^(44)^. These results suggest a higher burden and gender inequality in childcare in different households during school closure. Hence, social support for parents of school-age children is needed. In addition, some strategies to encourage children to manage circadian rhythms would be needed during school closure, such as having regular online meetings in the morning, although there is no established causal relationship between the temporal pattern of sleep and eating, lifestyle behaviours and dietary intake.

There are several limitations to the present study. First, our study sample was a convenient, not a nationally representative, sample. Possibility of selection bias needs to be recognised in this study. The study area was limited to only fourteen of the forty-seven prefectures in Japan. In addition, participants included in the analysis possibly had different characteristics from non-participants, although our sensitivity analysis with adjustment for household income and maternal education level did not change the direction of associations between LCA-derived classes and lifestyle behaviours and dietary intake. Thus, the generalisability of our results may be low. Second, the survey period might have affected the answers of participants, as the questionnaires were filled after schools reopened. Thus, participant answers did not fully reflect their lifestyle during school closure. However, four distinctive temporal patterns of sleep and eating were identified among participants. ‘Late’ and ‘Very late’ patterns might be rarely identified in a usual school year. This suggests that the effect of recall bias on the identification of patterns and the association between patterns and lifestyle variables might be low. Third, the variables used for analysis were all self-reported, and the validity of the questionnaires was insufficient or unknown. Previous studies have investigated the validity of the BDHQ15y against several biomarkers; the correlation coefficients were found to be low for all the examined nutrients^(27,28)^. The nonvalidated questions were used to assess sleep habits, physical activity, screen time and study time estimates. A previous systematic review found a high correlation between self-reported sleep time and those assessed with accelerometer among children^(45)^. Thus, analysing patterns of temporal sleep and eating time based on self-reported sleep habits could be acceptable. However, the questionnaire had the potential of measurement error resulting from recall bias and social desirability bias^(46,47)^. The parents of participating children possibly reported later wake-up time and earlier bedtime than the time when their children actually went to bed and woke up^(48,49)^. Thus, some participants were possibly misclassified. Regarding physical activity and sedentary behaviours, parents could overestimate their children’s physical activity and study time, while they underestimated screen time due to social desirability bias^(47)^. However, it was also possible that physical activity was possibly underestimated and sedentary behaviour was overestimated under the situation of the pandemic. Thus, it is unknown whether a possible measurement error overestimated or attenuated the associations between later timing of sleep and eating patterns and unfavourable lifestyle behaviour. Finally, we could not determine a causal relationship between temporal patterns of sleep and eating and lifestyle behaviours due to the cross-sectional study design. A sedentary lifestyle or longer screen time could have potentially adversely affected sleep habits.

In conclusion, more than half of the children in the present study had later circadian timings, especially for wake-up and eating breakfast and lunch. Later timings for sleeping and eating meals were associated with unfavourable lifestyle behaviours and dietary intake, including longer screen time and short study time, skipping meals, a lower consumption of vegetables and fruits and a higher consumption of sweetened beverages. Further studies are needed to investigate the environmental and social factors that determine differences in the temporal patterns of sleep and eating among children during school closure.

## Supplementary material

For supplementary material accompanying this paper visit https://doi.org/10.1017/S1368980022001148. click here to view supplementary material
