# Supplementary material for: Temporal patterns of sleep and eating among children during school closure in Japan due to COVID-19 pandemic: associations with lifestyle behaviours and dietary intake
Source: Public Health Nutr. 2022 May 16;26(2):393–407. doi: 10.1017/S1368980022001148 (PMC13076087; doi:10.1017/S1368980022001148)
Supplement: Supplementary file 1 [file S1368980022001148sup001.pdf]

	Breakfast		Lunch		Dinner		Late-night snacks	
	Frequency (times per week)	Time of eating (h)	Frequency (times per week)	Time of eating (h)	Frequency (times per week)	Time of eating (h)	Frequency (times per week)	Time of eating (h)
Example 1	7	6	7	12	7	19	0	-
Example 2	3	11	5	13	6	20	2	21
Example 3	6	11	6	14	7	20	1	22
Example 4	7	7	7	11	7	18	0	-
Example 5	6	10	4	12	5	17	5	21

⇩

	Generated binary variables																
	5-6 h	7 h	8 h	9 h	10 h	11 h	12 h	13 h	14 h	15 h	16 h	17 h	18 h	19 h	20 h	21 h	22-24 h
Example 1	1	2	2	2	2	2	1	2	2	2	2	2	2	1	2	2	2
Example 2	2	2	2	2	2	2	2	1	2	2	2	2	2	2	1	2	2
Example 3	2	2	2	2	2	1	2	2	1	2	2	2	2	2	1	2	2
Example 4	2	1	2	2	2	1	2	2	2	2	2	2	1	2	2	2	2
Example 5	2	2	2	2	1	2	2	2	2	2	2	1	2	2	2	1	2

Supplemental Fig. 1

Five examples of frequency and time of eating among participants and generated binary variables for latent class analysis

A. Three-class analysis

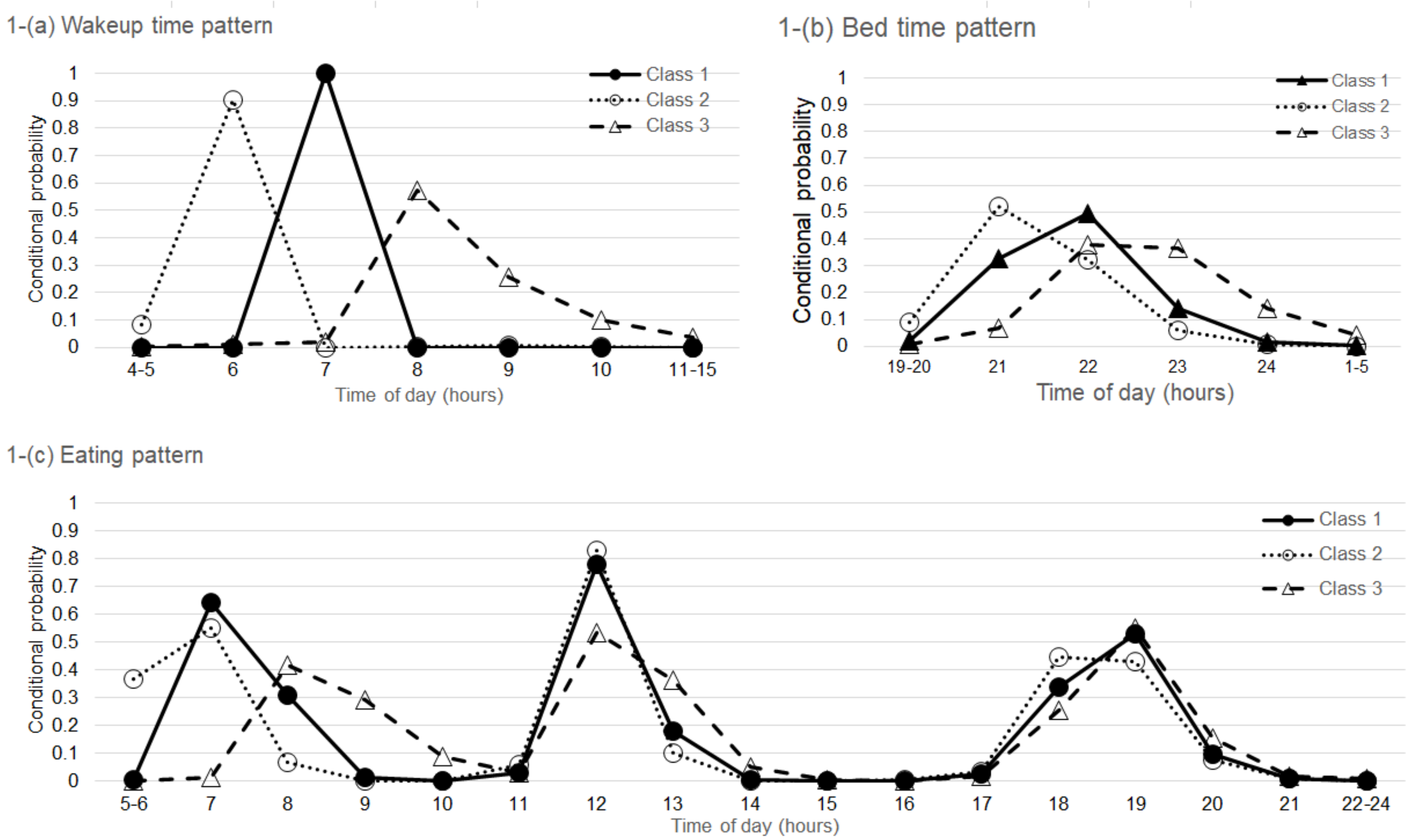

Class	Class 1	Class 2	Class 3
Proportion (%)	36%	20%	44%

Supplemental Fig. 2A
Conditional probabilities of (a) wake-up time, (b) bedtime, and (c) eating time across the day by according to three-class latent class analysis-derived temporal patterns of sleep and eating pattern classes among 6220 school-aged children

B. Four-class analysis

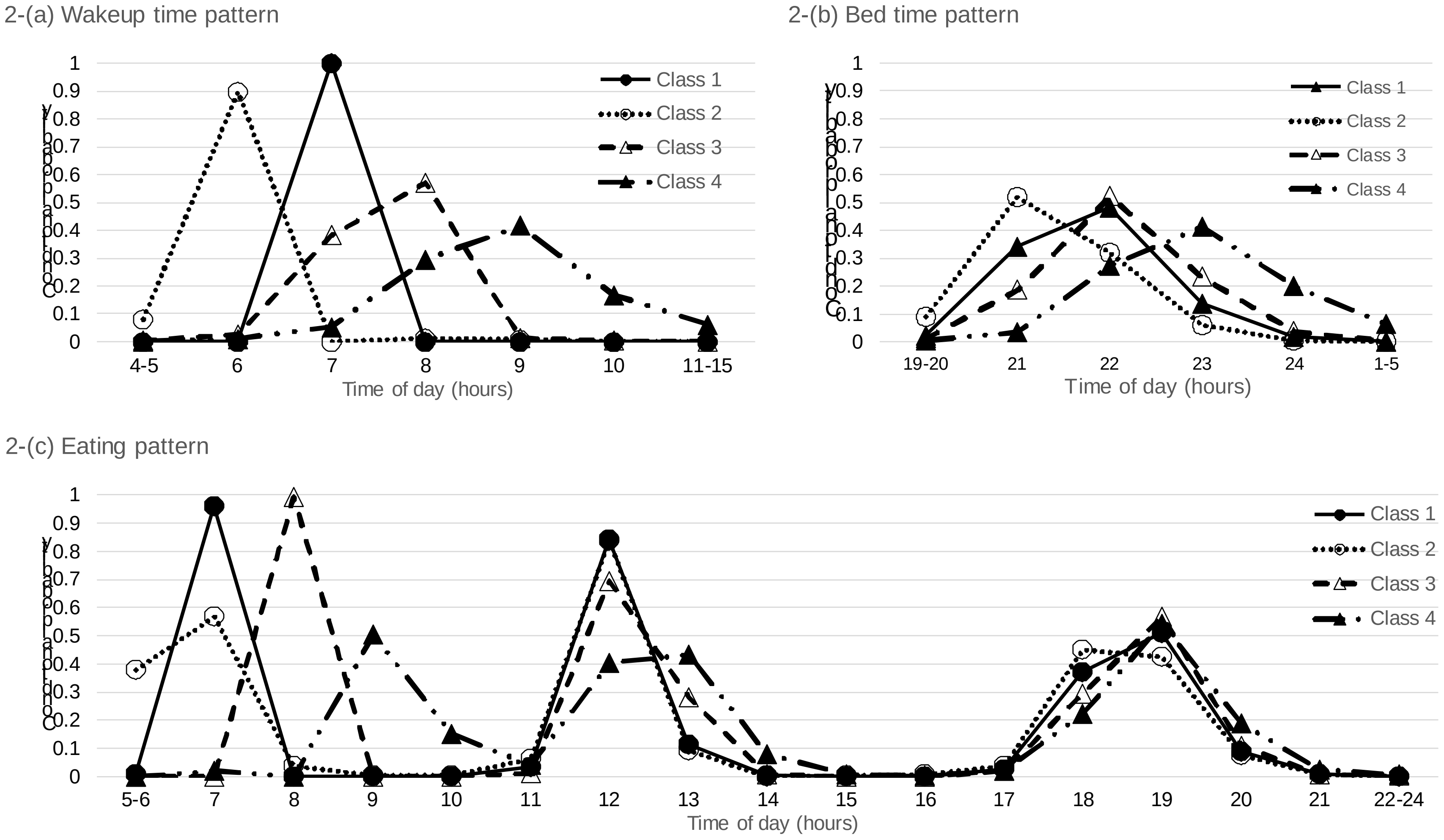

Class	Class 1	Class 2	Class 3	Class 4
Proportion (%)	24%	20%	30%	26%

Supplemental Fig. 2B

Conditional probabilities of (a) wake-up time, (b) bedtime, and (c) eating time across the day by according to four-class latent class analysis-derived temporal patterns of sleep and eating pattern classes among 6220 school-aged children

C. Five-class analysis

3-(a) Wakeup pattern

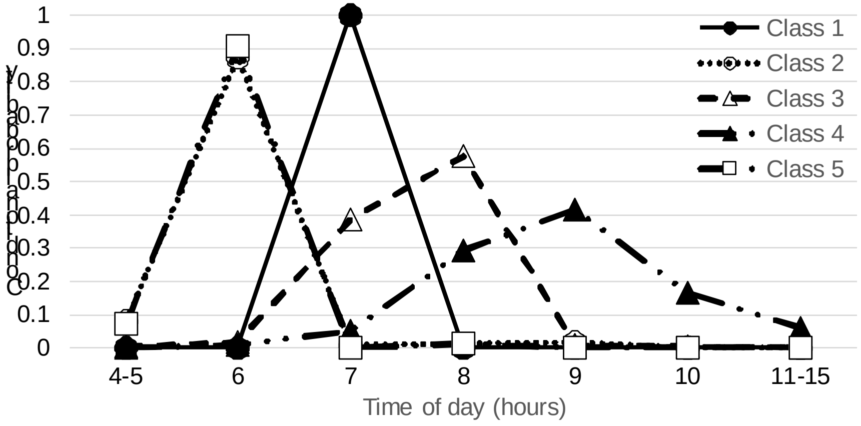

3-(b) Bed time pattern

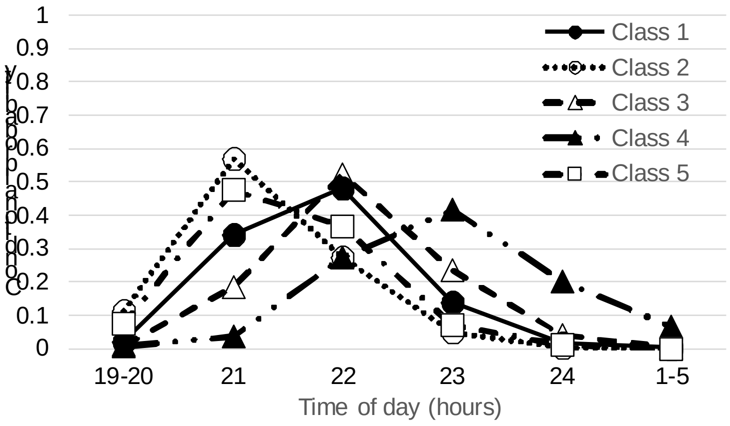

3-(c) Eating pattern

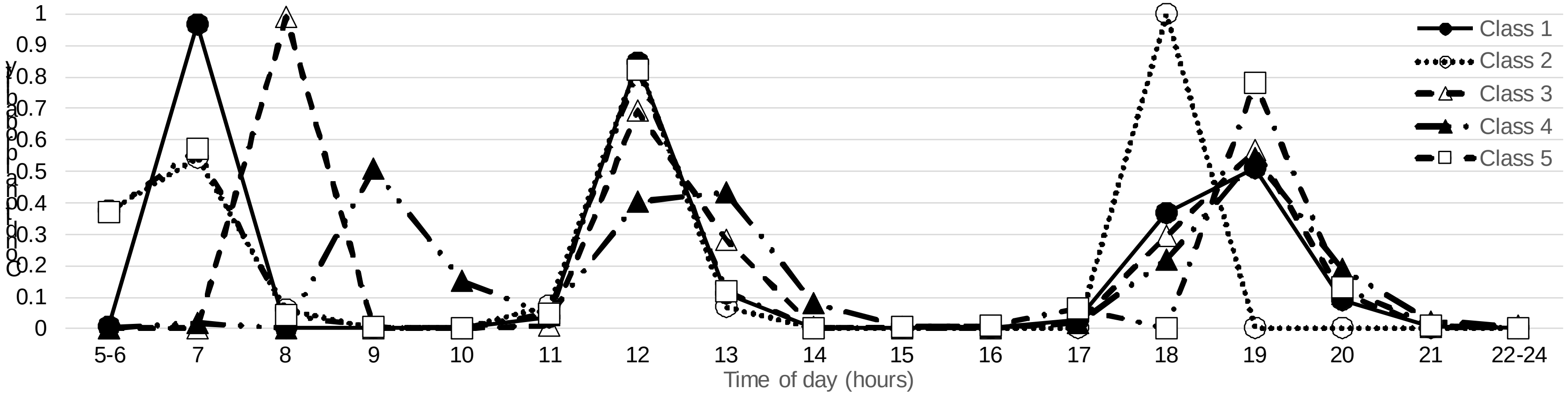

Class	Class 1	Class 2	Class 3	Class 4	Class 5
Proportion (%)	24%	9%	30%	26%	11%

Supplemental Fig. 2C

Conditional probabilities of (a) wake-up time, (b) bedtime, and (c) eating time across the day by according to five-class latent class analysis-derived temporal patterns of sleep and eating pattern classes among 6220 school-aged children

D. Six class analysis

4-(a) Wakeup time pattern

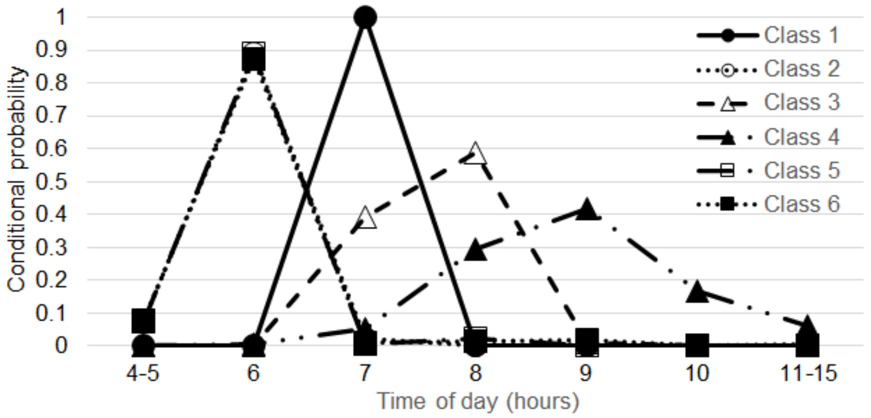

4-(b) Bed time pattern

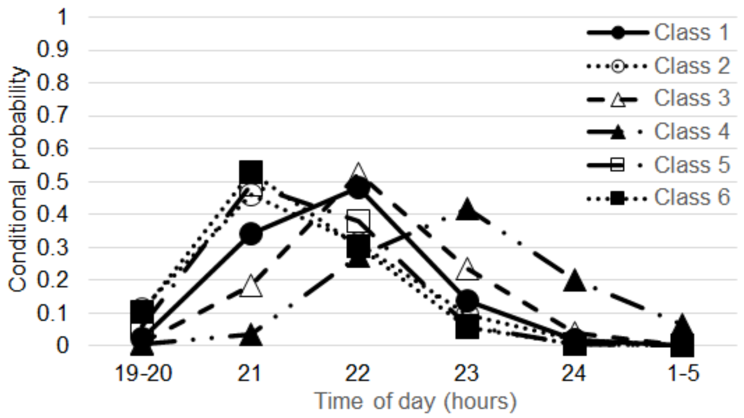

4-(c) Eating pattern

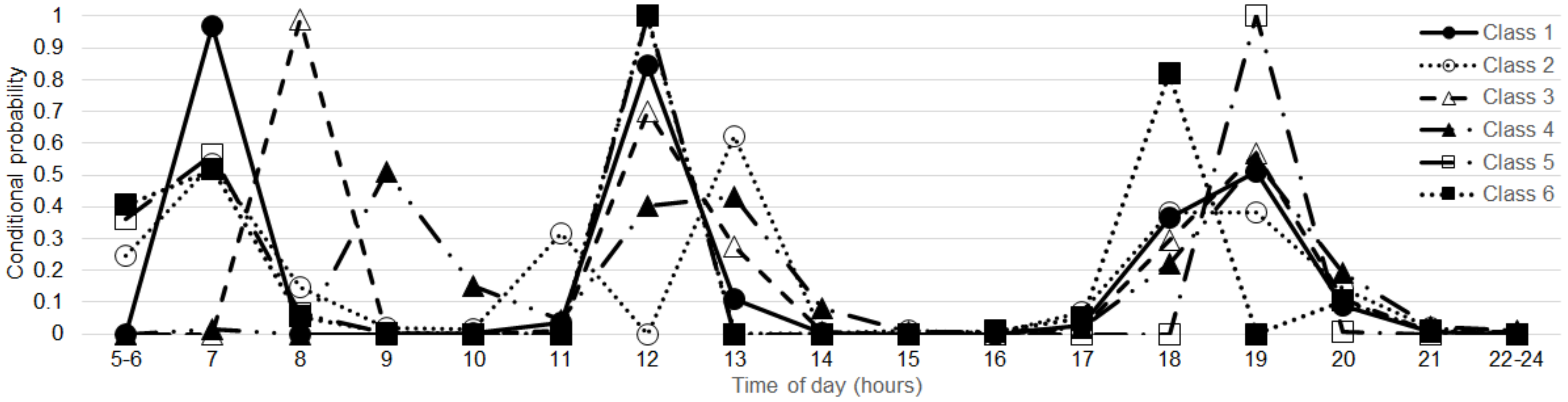

Class	Class 1	Class 2	Class 3	Class 4	Class 5	Class 6
Proportion (%)	24%	4%	29%	26%	8%	10%

Supplemental Fig. 2D

Conditional probabilities of (a) wake-up time, (b) bedtime, and (c) eating time across the day by according to six-class latent class analysis-derived temporal patterns of sleep and eating pattern classes among 6220 school-aged children

Supplemental Table 1. Lifestyle and dietary habits according to latent class analysis-derived temporal patterns of sleep and eating 6220 school-aged children (3rd to 6th grade of primary school and 1st to 3rd grade of secondary school)

	Very early pattern (n=1222)		Early pattern (n=1509)		Late pattern (n=1862)		Very late pattern (n=1590)		
	N	(%)	N	(%)	N	(%)	N	(%)	P*
Physical activity level†									<0.0001
Inactive	220	(18.0)	326	(21.6)	480	(25.8)	592	(37.2)	
Low	454	(37.2)	582	(38.6)	678	(36.4)	533	(33.5)	
Middle or High	545	(44.6)	595	(39.4)	710	(38.1)	505	(31.8)	
Screen time (h per day)‡									<0.0001
<2	325	(26.6)	330	(21.9)	328	(17.6)	140	(8.8)	
2-<4	515	(42.1)	681	(45.1)	791	(42.5)	494	(31.1)	
≥4	379	(31.0)	492	(32.6)	749	(40.2)	996	(62.6)	
Study time (h per day)§									<0.0001
<2	585	(47.9)	724	(48.0)	970	(52.1)	1095	(68.9)	
2-<4	480	(39.3)	581	(38.5)	709	(38.1)	451	(28.4)	
≥4	154	(12.6)	198	(13.1)	189	(10.2)	84	(5.3)	
Snack frequency (times per week)									<0.0001
0	71	(5.8)	62	(4.1)	83	(4.5)	89	(5.6)	
1	672	(55.0)	860	(57.0)	1072	(57.6)	791	(49.7)	
2	357	(29.2)	440	(29.2)	509	(27.3)	479	(30.1)	
≥3	122	(10.0)	147	(9.7)	198	(10.6)	231	(14.5)	

* P values were tested by $\chi 2$ test.

† Participants with physical activity level (PAL) <1.60(1) were categorised as "inactive or low PAL." PAL was calculated by dividing the metabolic equivalent-hour score by 24 h. The metabolic equivalent-hour score was estimated by summing the product of the time spent on each of a range of activities (sleeping, standing, walking, cycling, running, and other activities causing sweating) with various exercise intensities and metabolic equivalent values for each activity(2,3).

‡ Screen time included the time spent in watching television; using a computer, smartphone, or tablet; and playing video games.

§ Study time included time spent reading books and self-studying.

Supplemental Table 2. Adjusted dietary intake according to latent class analysis-derived temporal patterns of sleep and eating among 6220 school-aged children (3rd to 6th grade of primary school and 1st to 3rd grade of secondary school)

	Very early pattern (n=1219)		Early pattern (n=1503)		Late pattern (n=1868)		Very late pattern (n=1630)		P for trend*
	Mean	SE	Mean	SE	Mean	SE	Mean	SE	
Nutrient intake									
Protein (% of energy)	14.4	0.06	14.1	0.06	13.9	0.05	13.4	0.06	<0.0001
Total fat (% of energy)	30.8	0.16	31.0	0.14	30.7	0.13	30.9	0.14	0.27
Saturated fatty acids (% of energy)	10.1	0.07	10.2	0.06	10.0	0.06	10.2	0.06	0.33
Carbohydrate (% of energy)	53.4	0.18	53.4	0.16	53.9	0.15	54.1	0.16	0.002
Dietary fibre (g/4184 kJ)	6.0	0.04	5.9	0.04	5.8	0.04	5.4	0.04	<0.0001
Vitamin A (µg retinol equivalents/4184 kJ)	336	5.00	321	4.48	315	4.02	286	4.38	<0.0001
Thiamin (mg/4184 kJ)	0.41	0.002	0.40	0.002	0.40	0.002	0.39	0.002	<0.0001
Riboflavin (mg/4184 kJ)	0.75	0.005	0.73	0.004	0.71	0.004	0.68	0.004	<0.0001
Niacin (mg/1000kcal)	7.3	0.05	7.1	0.04	7.1	0.04	6.8	0.04	<0.0001
Vitamin B6 (mg/4184 kJ)	0.58	0.004	0.57	0.003	0.56	0.003	0.53	0.003	<0.0001
Vitamin B12 (µg/4184 kJ)	3.5	0.05	3.4	0.04	3.4	0.04	3.2	0.04	<0.0001
Folate (µg/4184 kJ)	170	1.50	165	1.35	161	1.21	147	1.32	<0.0001
Vitamin C (mg/4184 kJ)	56.3	0.65	55.6	0.58	54.9	0.52	50.0	0.57	<0.0001
Sodium (mg/4184 kJ)	1913	11.57	1910	10.37	1905	9.29	1879	10.13	0.08
Potassium (mg/4184 kJ)	1224	7.79	1191	6.98	1167	6.26	1107	6.82	<0.0001
Calcium (mg/4184 kJ)	344	3.09	330	2.77	318	2.48	304	2.70	<0.0001
Magnesium (mg/4184 kJ)	124	0.65	121	0.59	119	0.53	113	0.57	<0.0001
Iron (mg/4184 kJ)	4.0	0.02	3.9	0.02	3.8	0.02	3.6	0.02	<0.0001
Food intake (g/4184 kJ)									
Cereal	219	1.82	216	1.63	222	1.46	220	1.59	0.06
Sugars and confectioneries	49.4	0.80	52.1	0.72	51.6	0.65	55.3	0.70	<0.0001
Pulses	27.1	0.50	26.6	0.45	25.3	0.40	22.7	0.44	<0.0001
Vegetables	119	1.65	113	1.48	109	1.33	95	1.45	<0.0001
Fruits	30.2	0.81	29.9	0.73	28.7	0.65	24.6	0.71	<0.0001
Fish and shellfish	28.8	0.45	28.0	0.40	27.4	0.36	25.2	0.39	<0.0001
Meat	40.0	0.49	40.0	0.44	39.4	0.39	38.9	0.43	0.21
Dairy products	117	2.50	106	2.24	100	2.01	95	2.19	<0.0001
Sweetened beverages	30.4	1.95	38.5	1.75	39.5	1.57	58.5	1.71	<0.0001

* Trend of association was examined using a linear regression model with the ordinal scale of sleeping and eating pattern (1=”Very early” pattern, 2=”Early” pattern, 3=”Late” pattern, and 4=”Very late” pattern) as a continuous variable. Adjusted for sex, age, family structure, and sibling status.

Reference

1. Ministry of Health Labour and Welfare (2020) Dietary Reference Intakes for Japanese. (in Japanese).
2. Ainsworth BE, Haskell WL, Herrmann SD, et al. (2011) Compendium of physical activities: a second update of codes and MET values. *Med. Sci. Sports Exerc.* **43**, 1575–81.
3. Murakami K, Sasaki S, Okubo H, et al. (2007) Association between dietary fiber, water and magnesium intake and functional constipation among young Japanese women. *Eur. J. Clin. Nutr.* **61**, 616–622.
